# Hormonal Signaling during dPCD: Cytokinin as the Determinant of RNase-Based Self-Incompatibility in Solanaceae

**DOI:** 10.3390/biom13071033

**Published:** 2023-06-23

**Authors:** Ekaterina Zakharova, Tatiana Khanina, Andrey Knyazev, Natalia Milyukova, Lidia V. Kovaleva

**Affiliations:** 1 All-Russia Research Institute of Agricultural Biotechnology, 127550 Moscow, Russia; 2 Timiryazev Institute of Plant Physiology, Russian Academy of Sciences, 191186 Moscow, Russia

**Keywords:** RNase-based SI-induced PCD, caspase-like protease, cytokinin, actin cytoskeleton, pollen tube growth in vivo and in vitro

## Abstract

Research into molecular mechanisms of self-incompatibility (SI) in plants can be observed in representatives of various families, including Solanaceae. Earlier studies of the mechanisms of S-RNase-based SI in petunia (*Petunia hybrida* E. Vilm.) demonstrate that programmed cell death (PCD) is an SI factor. These studies suggest that the phytohormon cytokinin (CK) is putative activator of caspase-like proteases (CLPs). In this work, data confirming this hypothesis were obtained in two model objects—petunia and tomato (six Solanaceae representatives). The exogenous zeatin treatment of tomato and petunia stigmas before a compatible pollination activates CLPs in the pollen tubes in vivo, as shown via the intravital imaging of CLP activities. CK at any concentration slows down the germination and growth of petunia and tomato male gametophytes both in vitro and in vivo; shifts the pH of the cytoplasm (PHc) to the acid region, thereby creating the optimal conditions for CLP to function and inhibiting the F-actin formation and/or destructing the cytoskeleton in pollen tubes to point foci during SI-induced PCD; and accumulates in style tissues during SI response. The activity of the *ISOPENTENYLTRANSFERASE 5* (IPT5) gene at this moment exceeds its activity in a cross-compatible pollination, and the levels of expression of the *CKX1* and *CKX2* genes (*CK OXIDASE/DEHYDROGENASE*) are significantly lower in self-incompatible pollination. All this suggests that CK plays a decisive role in the mechanism underlying SI-induced PCD.

## 1. Introduction

Three main types of cell activities—division, extension, and programmed cell death (PCD)—determine the growth and development of an organism [1,2].

PCD in plants can vary but generally displays a set of common features [3]. In particular, the activity of caspase-like proteases, DNA fragmentation, the release of cytochrome c from mitochondria, cell shrinkage, the generation of reactive oxygen species, and the exposure of phosphatidylserine belong to such PCD manifestations [4].

Caspase-like proteases (CLPs) are the most typical PCD markers. CLPs are a group of endonucleases that cleave various proteins at aspartic acid residues [3]. These proteins normally reside in the central vacuole; however, the induction of PCD results in the degradation of this vacuole and the consequent release of CLPs and other lytic enzymes into the cytoplasm [5]. A considerable number of CLPs in the vacuole are inactive, and the activation of CLPs via autocatalysis is one of the early PCD events. This additional catalytic activity of CLPs makes them the main regulators of the other vacuolar proteases and the key regulators of PCD in plants [6,7,8].

Some cell types are able to initiate PCD as a result of intercellular signaling, in particular, in the case of a self-incompatibility (SI) response [3,9].

SI is a widespread genetic mechanism utilized by flowering plants to prevent inbreeding and enhance outcrossing. So far, over 100 plant families and approximately 40% of plant species use SI to avoid selfing [10,11]. However, only a few of them are studied, mainly the plants represented by Papaveraceae, Solanaceae, Rutaceae, Rosaceae, and Brassicaceae [11].

The results of robust in vivo functional assays for S-RNase and S-locus F-box protein (SLF) in *Petunia hybrida* E. Vilm. have made this species a good model system [12,13,14].

S-RNase, first discovered in *Nicotiana alata* [15], is the female determinant in the Solanaceae SI system. S-RNases act as highly specific cytotoxins and inhibit the growth of pollen tubes [16]. The SI of tomatoes is of an S-RNase type which is characteristic of Solanaceae [10]. The study of SI in tomatoes has been ongoing for a long time [17] and is still in progress [18,19,20]. Tomatoes are a popular object in the studies of SI evolution and variation in the S locus [18,21,22].

In the process of gametophytic SI of an S-RNase type in petunia, pollen tubes die in the pistil tissues 8 h after a self-incompatible pollination via S-RNase-based SI-induced PCD. This fact was discovered using different approaches, including the TUNEL (terminal deoxynucleotidyl transferase dUTP nick end labeling) assay, demonstrating DNA fragmentation in the self-incompatible pollen tubes growing in vivo [22].

Later, the activation of CLPs was demonstrated in the course of S-RNase-based SI-induced PCD in petunia [22]. In addition, the first preliminary data suggest that cytokinin (CK) is a potential CLP activator in this process [23].

The earlier data obtained in petunia [24,25,26,27,28,29] suggested hormonal signaling during a polarized growth of the pollen tube and its arrest as a result of SI; however, the mechanisms underlying the effect of phytohormones as signaling molecules in the pollen–pistil system are still vague.

It has been determined that CK decreases the PHc in the pollen tube to an acid range [30] and is able to inhibit F-actin formation, thereby destructing the cytoskeleton of the petunia pollen tube in vitro to F-actin point foci [29].

An analysis of our data suggests that in combination with CLPs and S-RNase, CK disorganizes the actin cytoskeleton of self-incompatible pollen tubes, thus interfering with the integrity of the membrane, destructing organelles and eventually degrading DNA as the final stage in the SI-induced PCD of *Petunia hybrida* E. Vilm.

Here, the question arises as to which is the primary factor, namely, the decreases in the pH of the cytoplasm caused by CK, which activates CLPs, or the destruction of the cytoskeleton in the pollen tube by CK, which triggers the enzyme activities in PCD.

In this study, we performed a set of experiments with the pollen tubes growing in vitro to elucidate the effects of (1) CK and (2) latrunculin B (Latr B), an inhibitor of F-actin polymerization), treatments on CLP activity in the in vitro growing pollen tubes.

The experimental scheme was expanded. The study involved two objects with the addition of the tomato, another member of the Solanaceae family. For this study, we selected self-incompatible species of wild tomatoes, *Solanum pennellii*, *S. habrochaites*, and *S. chilense*, and the self-compatible *S. lycopersicum* cultivar Blush.

Correspondingly, the goal of this work was to test the above-stated hypothesis, focusing on the interaction between CK and the cytoskeleton (the hormone-induced remodeling of the cytoskeleton). The intravital CLP imaging allowed us to determine the particular cells in pollen tubes and/or the surrounding pistil tissues where CLPs were activated. We investigated the regulation of CK biosynthesis at the time of the self-incompatibility reaction in petunias (*IPT5*, *LOG*, *CKX1*, and *CKX2* genes).

The activity of the *IPT5* (*ISOPENTENYLTRANSFERASE 5*) gene at this moment of growth arrest for the self-incompatible pollen tubes exceeds its activity in compatible pollination, and the levels of expression of *CKX1* and *CKX2* (*CK OXIDASE/DEHYDROGENASES*) genes are significantly lower in self-incompatible pollination; the inhibitory effect of CK on pollen tube growth and actin polymerization and the opposite stimulatory effect on caspase-like activity that we have identified suggest that CK plays a key role in the arrest of pollen tube growth during S-RNase-based, SI-induced PCD in Solanaceae.

## 2. Materials and Methods

### 2.1. Plant Material and Growing Conditions

Six Solanaceae representatives were used in the work:

(1) Two clones (self-compatible and self-incompatible) of the petunia *P. hybrida;*
(2) Four tomato species of the genus *Solanum* (one self-compatible cultivated tomato *S. lycopersicum* cultivar Blush and three self-incompatible tomato species *S. chilense*, *S. pennellii*, and *S. habrochaites*).

The *P. hybrida* plants vegetatively propagated from two clones (self-compatible and self-incompatible) from the laboratory collection were grown in soil in a greenhouse with natural illumination. The tomato plants were grown from seed in soil under the same conditions.

The seeds of three species of wild, self-incompatible tomatoes, *S. chilense*, *S. pennellii* (Rio Atico, Km 61 Arequipa, Peru), *S. habrochaites* (Rio Casma, Ancash and (Minas de Acari, Arequipa, Peru), were obtained from the C.M. Rick Tomato Genetics Center, University of California, Davis, United States. The cultivated tomato *S. lycopersicum* cultivar Blush (Partner Company, Moscow, Russia) was used as a self-compatible sample; this cultivar has a long flowering period and a high fruit yield.

### 2.2. Treatments

#### 2.2.1. In Vitro

The freshly harvested pollen grains of two clones of petunia and four species of tomato were cultivated for 3 h in a thermostat at a temperature of 25 °C on a liquid aqueous medium containing 0.4 M of sucrose and 1.6 mM of H_3_BO_3_. Pollen (2 mg) and culture medium (2 mL) were placed into a vial and supplemented with zeatin or Latr B simultaneously with the culture medium.

In the in vitro experiments, zeatin was used at doses of 10^−3^, 10^−6^, 10^−9^, or 10^−12^ and 5 nM of Latr B.

The degree of germination was assessed according to the number of germinated pollen grains randomly selected and examined over four microscope (Zeiss Axioplan, Carl Zeiss, Germany) fields (*n* = 200) on an hourly basis. The lengths of the pollen tubes were measured using the AxioVision 4.8 (Carl Zeiss, Germany) software.

#### 2.2.2. In Vivo

**Figure 1:**
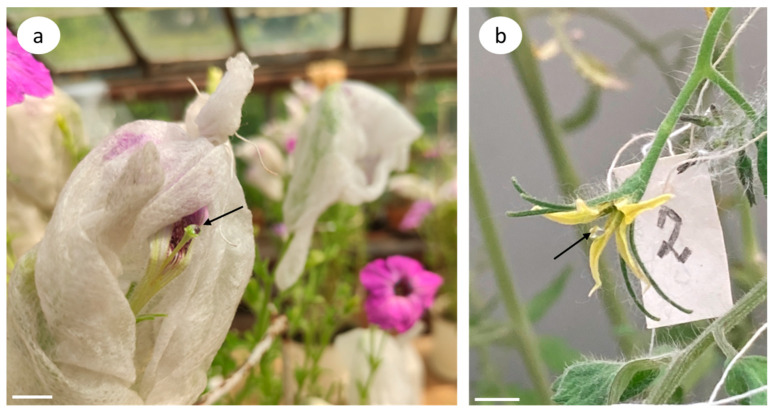
Zeatin-treated flowers of (**a**) petunia and (**b**) tomato cultivar Blush on the next day after emasculation. Arrow denotes a drop of zeatin solution on the stigma. Bar, 1 cm.

Petunia is an ideal object for studying the reproductive process. It has large flowers with a pistil length of 5–7 cm (Figure 1a) and abundant flowering (all year round under controlled conditions).

However, tomato flowers are considerably more difficult objects since the flowers and reproductive organs are three–fourfold smaller when compared with petunia (Figure 1b).

Both petunia and tomato have wet stigmas with abundant exudate. The Solanaceae representatives have a two-phase exudate comprising a hydrophobic phase (upper layer) enriched with lipids, with a hydrophilic phase (a very thin aqueous layer) below. A pollen grain falling through the upper layer directly contacts stigma cells and germinates after the stages of adhesion and rehydration [31].

The stigmas of the flowers emasculated the day before were pretreated with CK (zeatin) or Latr B (inhibitor of F-actin polymerization) 2 h before pollination. A zeatin solution (5 µL of a 10 µM solution) or Latr B (5 nM) was pipetted onto the stigmas of petunia; in the case of tomato, 1.5 µL of a 10 µM zeatin solution was used or similarly, 5 nM of Latr B was used. In the control variants, a drop of water was applied (Figure 1).

The stigmas of the self-incompatible petunia variant were pollinated with its own pollen (self-incompatible pollination) or with the pollen of a self-compatible petunia variant (cross-compatible pollination).

The self-compatible and self-incompatible tomato plants emasculated and isolated the day before flower opening were self-pollinated with the pollen harvested from opened flowers.

The pollinated pistils were harvested 2, 4, 6, and 24 h after the pollination and were fixed in acetic alcohol (3:1). Parts of the pollinated pistils were left for the control of seed setting.

### 2.3. Imaging of Growing Pollen Tubes

#### 2.3.1. In Vitro

The degree of germination was assessed according to the number of germinated pollen grains randomly selected and examined over four microscope (Zeiss Axioplan, Carl Zeiss, Jena, Germany) fields (*n* = 200) on an hourly basis. The length of pollen tubes was measured using the AxioVision 4.8 (Carl Zeiss, Germany) software.

#### 2.3.2. In Vivo (in Pistil Tissue)

The growing pollen tubes were imaged via aniline blue staining based on the ability of its fluorochrome to bind to callose, which is contained in the pollen grain and pollen tube cell wall.

The pollinated petunia and tomato pistils fixed with acetic alcohol (90% ethanol and acetic acid solution at a ratio of 3:1) were used in the experiments. The pistils were macerated with a 20% KOH alcohol solution for 20–40 min, rinsed twice with distilled water, and stained with a 0.01% aniline blue solution for 30–40 min. The stained pistils were placed onto glass slides in a drop of glycerin mixed with water (1: 1), sealed with a cover glass, slightly squashed, and examined using a Zeiss Axioplan (Carl Zeiss, Germany) fluorescence microscope with an excitation filter of 365 nm and an emission filter of 420 nm. At least 200 pollen tubes were measured in each experiment.

### 2.4. Imaging of CLP Activity in Growing Pollen Tubes via Fluorescence Technique

#### 2.4.1. In Vivo

The CLP activity in living pollen tubes was visualized using the Image-iT™ LIVE Green Poly Caspases Detection Kit (Invitrogen, Thermo Fisher Scientific, United States) with a FLICA reagent for the detection of most caspases (including caspases 1 and 3–9). It also contains propidium iodide, the dye that allows for the concurrent assessment of the nuclear morphology and the integrity of the plasma membrane. The protocol was modified for the pollen–pistil system.

We sampled the pistils of petunia and tomato over 2 h after compatible pollinations (pretreated with 10 µM of zeatin or 5 nM of Latr B (control-drop of water) 2 h before pollination). Very thin longitudinal sections (4 mm long) of pistil (stigma and the top of style) were obtained and labeled according to the manufacturer’s protocol. The sections were placed into 1.5 mL Eppendorf tubes; immediately supplemented with the working solution, the FAM-VAD-FMK poly caspases reagent, a FLICA reagent, in PBS (pH 7.4) to completely cover the sections; and incubated at ambient temperature in the dark for 60 min. The sections were then gently removed from the FAM-VAD-FMK poly caspases reagent, immersed in 5 mM of propidium iodide in PBS (pH 7.4), incubated at an ambient temperature in the dark for 10 min, rinsed twice with a wash buffer, and placed into an apoptosis fixative solution (10% formaldehyde solution). Fixation makes it possible to examine a sample over 24 h. The samples were placed on a microscope slide in a drop of wash buffer, covered with a coverslip, and slightly crushed. The pistil sections were imaged using a fluorescence microscope with 488/530 nm (FAM-VAD-FMK poly caspases reagent) and 535/617 nm (propidium iodide) filter sets. The pictures were taken with a THUNDER 3D Tissue microscope using a DFT51111 filter set, an LED3 fluorescence light source, and a DFC9000 GTC digital camera (Leica Microsystems, Wetzlar, Germany). Multichannel fluorescence recording, image processing for brightness/contrast, and color settings were performed using LasX software (Leica Microsystems, Wetzlar, Germany). For each variant of pollination, five individual pistils were stained and imaged. The green fluorescence signal is a direct measure of the amount of an active caspase that was present at the moment when the reagent was added. This signal in the pollen tubes indicated the presence of CLP activities in the cell. In each variant of the experiment, at least 200 pollen tubes were examined.

#### 2.4.2. In Vitro

The petunia and tomato pollens were grown in culture media for 1.5 h. The pollen tubes were harvested via filtration though a net (mesh <10 µm), transferred to the medium containing zeatin (10 µM) or Latr B (5 nM), kept for 1 h under the same conditions, and placed in the corresponding solutions of the Image-iT™ LIVE Green Poly Caspases Detection Kit used, according to the protocol (see Section 2.4). After fixation, a drop of the solution with pollen was placed on a glass slide, sealed with a cover glass, and examined using a fluorescence microscope. In each variant of the experiment, at least 200 pollen tubes were examined.

### 2.5. RT-qPCR

To determine the expression of isopentenyltransferase 5 (*IPT5*) (Peaxi162Scf00016g00945, Solanaceae Genomics Network), we used a reverse transcription quantitative PCR (RT-qPCR). The total RNA of the pollinated petunia pistils (stigma and style) 7 h after pollination (in three biological replicates) was isolated using ExtractRNA (Evrogen, Moscow, Russia). The RNA quality and quantity were evaluated via electrophoresis in agarose gel with ethidium bromide staining. The cDNA for the RT-qPCR was synthesized using an M-MLV RT kit (Evrogen, Moscow, Russia) according to the manufacturer’s protocol, employing oligo(dT)17-primers (Table 1) from 2 μg of total RNA after DNase treatment. The primers were designed using Primer-BLAST [32]. The primers of CK oxidase/dehydrogenases (*CKX1* and *CKX2*) and CK riboside 5′-monophosphate phosphoribohydrolase (*LOG*) were used from previously published research [33]. The minus reverse transcriptase control (–RT) contained RNA without reverse transcriptase treatment to confirm the absence of DNA in the samples. The qPCR was performed using the qPCRmix-HS SYBR 5X (Evrogen, Moscow, Russia) on a Real-time CFX96 Touch (Bio-Rad, United States) in three biological and three technical replicates. The representation of cDNA was normalized using the stably transcribed reference gene elongation factor 1α (SGN-U207468, Solanaceae Genomics Network) [34]. The reaction comprised one cycle of 95 °C for 3 min, 40 cycles of 95 °C for 15 s, and final stage of 65 °C for 40 s. The data were analyzed via the ^2−ΔΔCT^ method to reflect the relative gene expression levels [35].

### 2.6. Statistics

The experiments were performed in three–five biological replicates. The statistical significance was assessed using Student’s *t*-test (*p* ≤ 0.05); the data are shown as means and their standard deviations.

## 3. Results

### 3.1. Effect of Zeatin on In Vivo Pollen Tube Germination and Growth in Tomato

**Figure 2:**
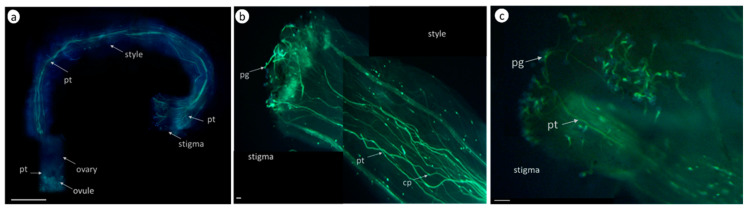
Pollen tube growth in vivo in the pistil tissues of *S. lycopersicum* cultivar Blush; aniline blue staining (365/420 nm); pg, pollen grain; pt, pollen tube; cp, callose plug. (**a**) Pollen tube growth in vivo (control) 24 h after self-compatible pollination, pollen tubes reached the ovary. Bar size, 1 mm. (**b**) Pollen tube growth in vivo (control) 4 h after self-compatible pollination. Bar size, 100 µm. (**c**) Zeatin (10 µM) pretreatment 2 h before pollination. 4 h after self-compatible pollination. Bar size, 100 µm.

The self-pollinated pollen of self-compatible *S. lycopersicum* cultivar Blush germinates on the stigma, grows along the style’s conducting tissues, and reaches the ovary in 24 h with subsequent fertilization (Figure 2a,b, Table 2).

The zeatin (10 µM) pretreatment of *S. lycopersicum* cultivar Blush stigmas 2 h before pollination considerably inhibited the growth of pollen tubes (Figure 2c), interfering with seed setting. Table 2 lists the corresponding results.

In the case of the self-pollination of the self-incompatible tomatoes *S. chilense*, *S. habrochaites*, and *S. pennellii*, almost all pollen grains that reached the stigma germinated; the pollen tubes grew along the style’s conducting tissues for 2–4 h and stopped.

**Figure 3:**
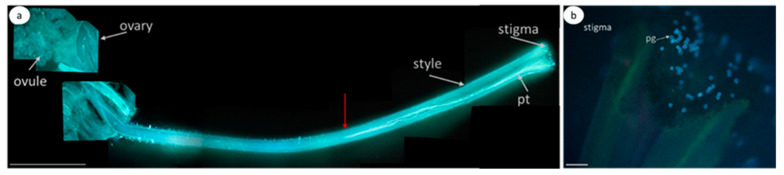
Pollen tube growth in vivo in the pistil tissues (self-incompatible tomato *S. chilense*). Aniline blue staining (365/420 nm); pg, pollen grain; pt, pollen tube. (**a**). Pollen tube growth in vivo (control): self-incompatible pollination. Pollen tubes stopped growing at a distance of 1.5–2 mm from the stigma surface. Red arrow denotes the point of pollen tube growth arrest. Bar size, 1 mm. (**b**). Zeatin (10 µM) pretreatment 2 h before pollination. No pollen germination. Bar, 100 µm.

The pollen tubes of *S. chilense* stopped at a distance of 1.5–2 mm from the stigma surface owing to the function of the S-RNase-based SI mechanism and remained at the same level at 24 h (Figure 3a, Table 3).

The zeatin (10 µM) pretreatment of *S. chilense* stigmas 2 h before pollination arrested both the germination and growth of the pollen tubes (Figure 3b, Table 3). The pollen grains failed to germinate, and the seeds did not set. The results for *S. pennellii* and *S. habrochaites* were similar.

Thus, the exogenous zeatin (10 µM) pretreatment of stigmas slowed down the pollen tube growth in a self-compatible pollination of *S. lycopersicum* cultivar Blush and arrested pollen germination in self-incompatible pollination in *S. chilense*, *S. habrochaites*, and *S. pennellii*.

### 3.2. Effect of Zeatin Treatment on Caspase-like Protease Activities in the Pollen Tubes (P. hybrida and S. lycopersicum Cultivar BLUSH) Growing In Vivo

#### 3.2.1. *P. hybrida* of Self-Incompatible Clone and Cross-Compatible Pollination

**Figure 4:**
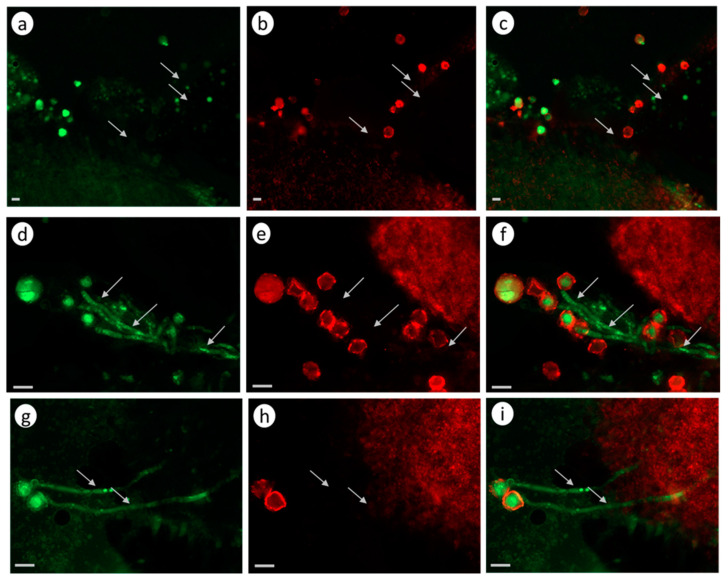
CLP activity in the petunia pollen tubes growing in vivo (2 h after pollination). Fluorescence microscopy of the pollen tubes growing in vivo using an Image-iT™ LIVE Green Poly Caspases Detection Kit. Arrows denote pollen tubes or the sites where they are located; bar, 50 µm. (**a**–**c**). Cross-compatible pollination (control, without zeatin pretreatment). The absence of green fluorescence signal in pollen grains and tubes indicates the absence of CLP activity in cells. (**d**–**f**). Self-incompatible pollination (control, without zeatin pretreatment). The green fluorescence signal in the pollen grains and tubes indicates the presence of CLP activity in cells. (**g**–**i**). Cross-compatible pollination (+zeatin pretreatment). The green fluorescence signal in the pollen grains and tubes indicates the presence of CLP activity in cells. (**a**,**d**,**g**). Detection of CPL activity (488/530 nm FAM-VAD-FMK poly caspases reagent). (**b**,**e**,**h**). Propidium iodide (535/617 nm). (**c**,**f**,**i**). FAM-VAD-FMK poly caspases reagent + propidium iodide.

In the case of self-compatible pollination (control without pretreatment; Figure 4a–c), any green fluorescence in the pollen tubes is unobservable, suggesting that the CLPs are inactive.

In the control self-incompatible pollination of petunia (Figure 4d–f), we observe a characteristic bright green fluorescence signal. This is another demonstration that the PCD enzymes actively work as early as 2 h after self-incompatible pollination. 

In the experimental variant (zeatin pretreatment), a bright green fluorescence signal is observed (Figure 4g–I, Video S1), demonstrating the presence of active CLPs in the petunia pollen tubes at this moment (2 h after cross-compatible pollination).

However, propidium iodide (a component of the Image-iT™ LIVE Green Poly Caspases Detection Kit) cannot enter pollen tubes and stain the nuclei red, demonstrating that the pollen tubes are still living, although the PCD process has already been triggered.

#### 3.2.2. *S. lycopersicum* Cultivar Blush

**Figure 5:**
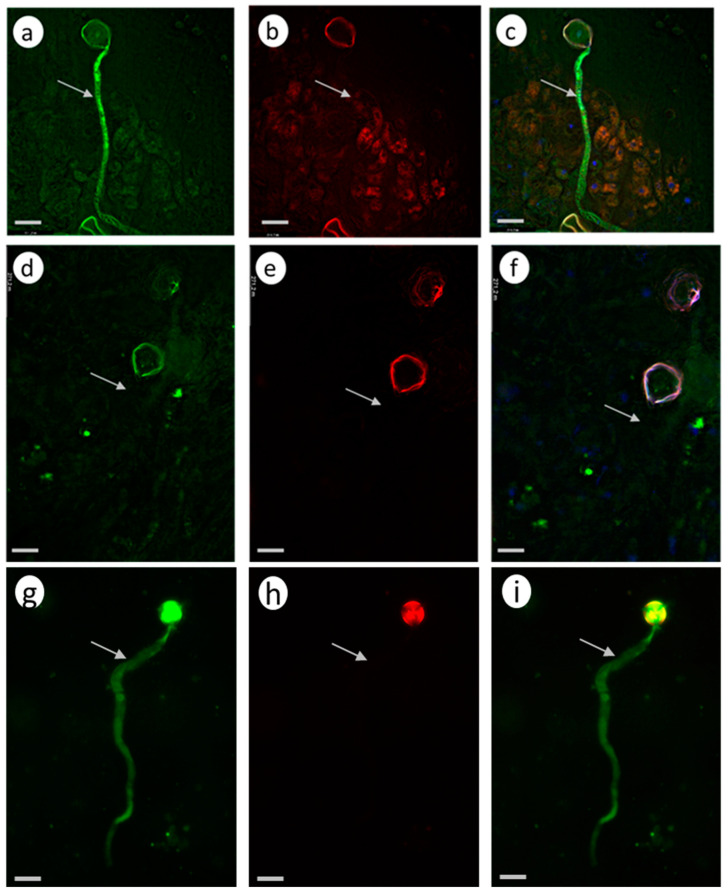
CLP activity in the tomato pollen tubes growing in vivo (2 h after self-compatible pollination). Fluorescence microscopy of the pollen tubes growing in vivo using an Image-iT™ LIVE Green Poly Caspases Detection Kit. Arrows denote pollen tubes or the sites where they are located; bar, 10 µm. (**a**–**c**). Cross-compatible pollination + zeatin (10 µM) pretreatment 2 h before pollination. The green fluorescence signal in the pollen grains and tubes indicates the presence of CLP activities in the cells; (**d**–**f**). Self-compatible pollination (without zeatin pretreatment). The absence of green fluorescence signal in pollen grains and tubes indicates the absence of CLP activity in cells. (**g**–**i**) Self-incompatible pollination *S. chilense* (control, without zeatin pretreatment). The green fluorescence signal in the pollen grains and tubes indicates the presence of CLP activity in cells. (**a**,**d**,**g**). Detection of CPL activity (488/530 nm FAM-VAD-FMK poly caspases reagent). (**b**,**e**,**h**). Propidium iodide (535/617 nm). (**c**,**f**,**i**). FAM-VAD-FMK poly caspases reagent + propidium iodide.

The zeatin (10 µM) pretreatment of the stigmas of the emasculated tomato *S. lycopersicum* cultivar Blush flowers 2 h before compatible pollination activated CLPs (Figure 5a–c). In this case, the green fluorescence signal is observed versus the control variant (compatible pollination without pretreatment; Figure 5d–f), in which this signal is unobservable. In the control self-incompatible pollination of tomato *S. chilense* (Figure 5g–i), we observe a characteristic bright green fluorescence signal. 

Thus, the zeatin (10 µM) pretreatment of the petunia and tomato stigmas 2 h before pollination activates CLPs.

### 3.3. Effect of Zeatin on Pollen Tube Germination and In Vitro Growth in Tomato

**Figure 6:**
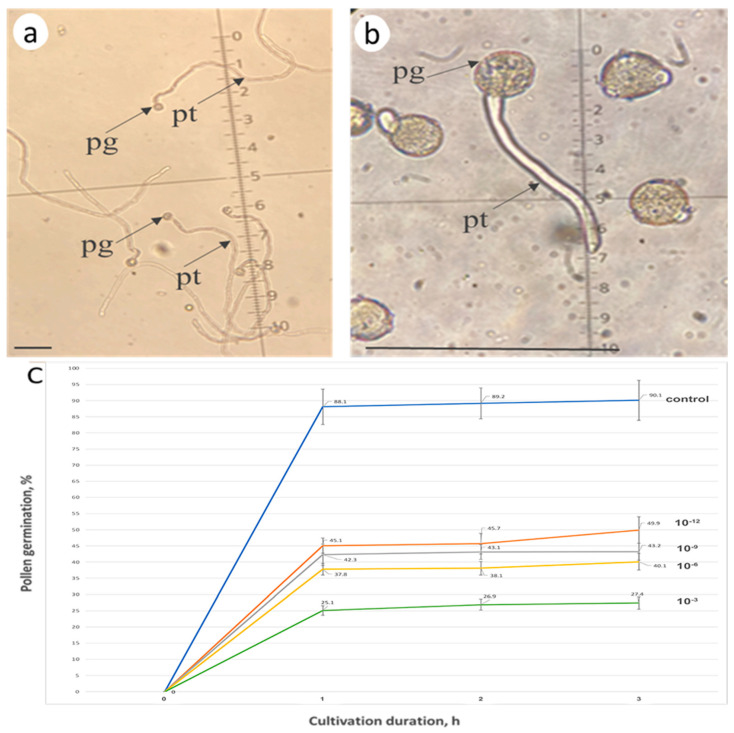
Effect of zeatin treatment on the in vitro pollen tube germination (3 h of cultivation): (**a**) control (without zeatin); (**b**) medium with zeatin (10^−6^); and (**c**) pollen germination rate (%) on the culture medium containing zeatin at different concentrations; pg, pollen grain; pt, pollen tube; bar, 100 µm.

The effect of the addition of zeatin to the medium for in vitro pollen cultivation was unambiguous. At all concentrations used (10^−3^, 10^−6^, 10^−9^, and 10^−12^), zeatin inhibited the in vitro germination of pollen grains of all four tomato species (*S. pennellii*, *S. habrochaites*, *S. chilense*, and *S. lycopersicum* cultivar Blush) used in the experiments (Figure 6).

Thus, the addition of zeatin (10^−3^, 10^−6^, 10^−9^, and 10^−12^) to the culture medium had an inhibitory effect at all tested concentrations. A distinct dependence on the concentration of this phytohormone was observable, being the least pronounced at the lowest zeatin concentration (10^−12^).

### 3.4. Effect of Zeatin on CLP Activities in the Pollen Tubes of Compatible Petunia Clone and S. lycopersicum Cultivar Blush Growing In Vitro

The tomato and petunia pollen grains were cultivated on the medium containing 0.4 M of sucrose and 1.6 mM of H_3_BO_3_ for 1.5 h. By that time, the length of the tomato pollen tubes was 89 ± 4.6 µm and the length of the petunia pollen tubes was 123 ± 8.1 µm. Then, the pollen grains with growing pollen tubes were transferred to the medium with zeatin (10 µM) for 1 h. The length of the pollen tubes did not change over this 1 h cultivation.

**Figure 7:**
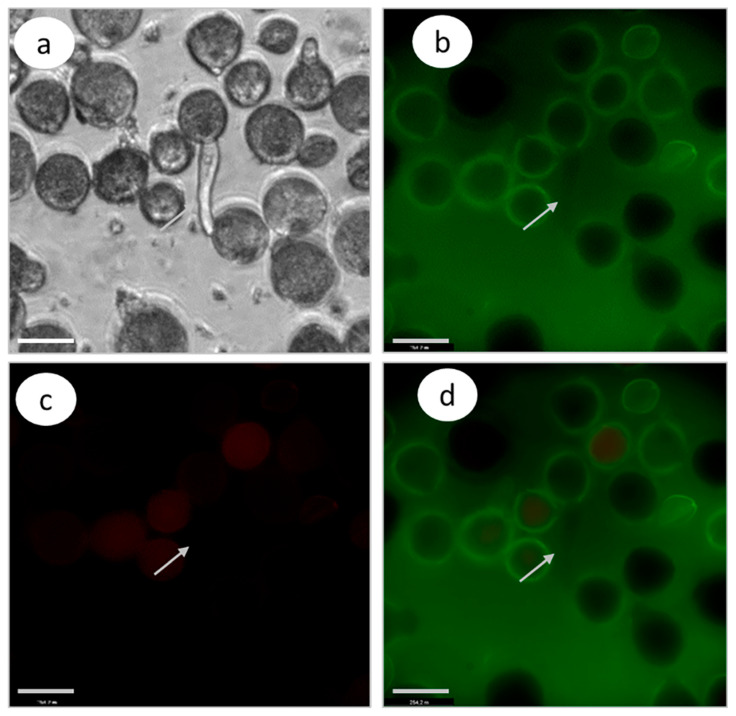
CLP activity in the in pollen tubes of the tomato *S. lycopersicum* cultivar Blush growing in vivo and treated with zeatin (10 µM); fluorescence microscopy using Image-iT™ LIVE Green Poly Caspases Detection Kit. The absence of green fluorescence signal in pollen grains and tubes indicates the absence of CLP activity in cells (arrows denote the location of pollen tube; bar, 10 µm): (**a**) light microscopy; (**b**) detection of caspase activities (488/530 nm FAM-VAD-FMK poly caspases reagent); (**c**) propidium iodide (535/617 nm); and (**d**) FAM-VAD-FMK poly caspases reagent + propidium iodide.

A further analysis of CLP activities according to the Image-iT™ LIVE Green Poly Caspases Detection Kit demonstrated the absence of CLP activities. A green fluorescence signal was absent in both cases—the *S. lycopersicum* cultivar Blush (Figure 7) and the compatible petunia clone. 

### 3.5. Effect of Latrunculin B (Inhibitor of F-Actin Polymerization) Treatment on the Caspase-like Protease Activities in Petunia and Tomato Growing Pollen Tubes

#### 3.5.1. In Vivo

**Figure 8:**
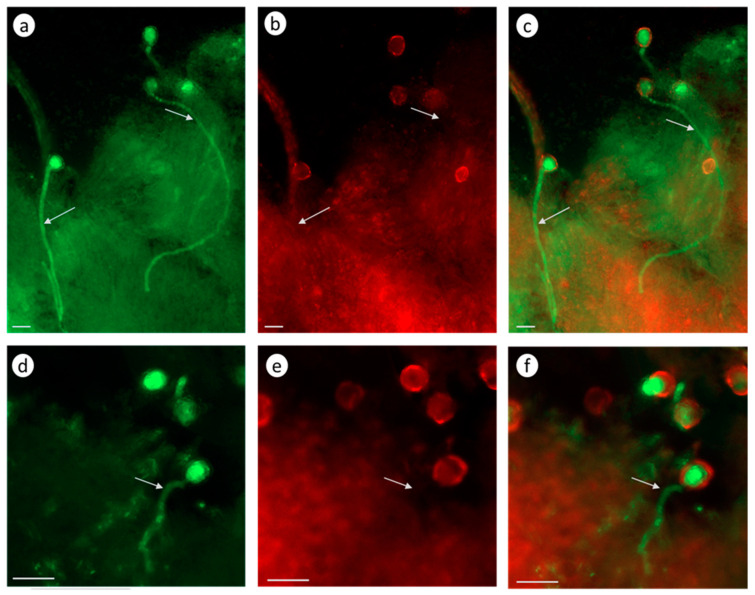
CLP activity in the pollen tubes growing in vivo (2 h after self-compatible pollination); fluorescence microscopy using Image-iT™ LIVE Green Poly Caspases Detection Kit. The green fluorescence signal in the pollen grains and tubes indicates the presence of CLP activities in cells (arrows denote pollen tubes; bar, 50 µm). (**a**–**c**). Effects of Latr B (5 nM) treatment of the stigmas on CLP activity in the petunia pollen–pistil system after a cross-compatible pollination. (**d**–**f**). Effects of Latr B (5 nM) treatment of the stigmas on CLP activity in the tomato S. lycopersicum cultivar Blush pollen–pistil system after a self-compatible pollination. (**a**,**d**). Detection of CPL activity (488/530 nm FAM-VAD-FMK poly caspases reagent). (**b**,**e**). Propidium iodide (535/617 nm). (**c**,**f**). FAM-VAD-FMK poly caspases reagent + propidium iodide.

The conditions and arrangement of the experiment with Latr B were the same as with the zeatin treatment. The stigmas of the emasculated tomato and petunia flowers treated with Latr B (5 nM) 2 h before pollination activated PLCs. Green fluorescence signals were observable in both petunia (Figure 8a–c) and tomato (Figure 8d–f) pollen tubes.

An analysis of these results suggests that the destruction of the actin cytoskeleton by Latr B treatment in the in vivo growing petunia and tomato pollen tubes activates CLPs.

#### 3.5.2. In Vitro

The conditions and arrangement of the experiment with Latr B were the same as with the zeatin treatment of the in vitro growing pollen tubes (see Section 3.4). After their germination in culture medium for 1.5 h, the pollen grains with pollen tubes were transferred to the medium supplemented with Latr B (5 nM) and left for further germination for 1 h. Similar to the experiment with zeatin, the lengths of the pollen tubes on the medium with Latr B did not change, and the growth of pollen tubes was arrested.

**Figure 9:**
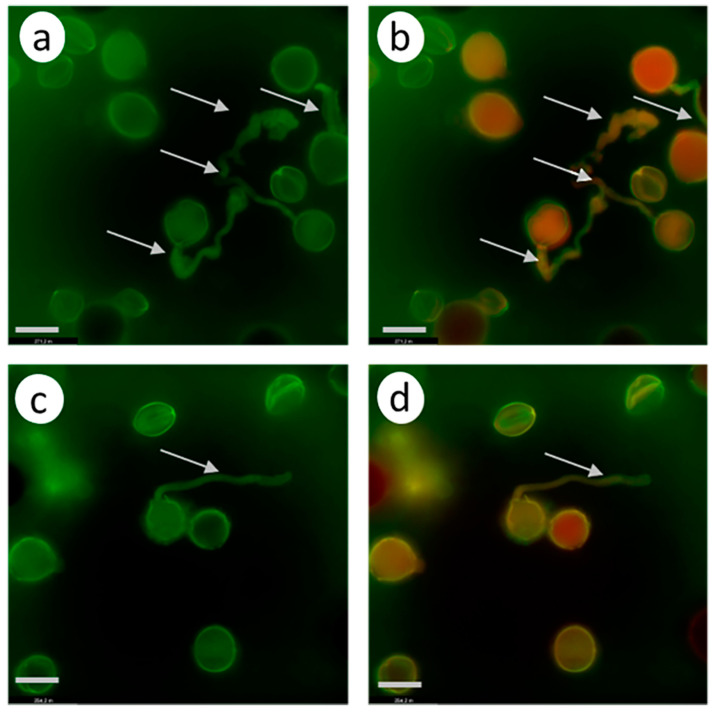
CLP activity in the in vitro growing pollen tubes of (**a**,**b**) petunia and (**c**,**d**) tomato after Latr B (5 nM) treatment. The green fluorescence signal in the pollen grains and tubes indicates the presence of CLP activities in cells (arrows denote pollen tubes): (**a**,**c**) the detection of CPL activity (488/530 nm FAM-VAD-FMK poly caspases reagent); (**b**,**d**) FAM-VAD-FMK poly caspases reagent + propidium iodide; (**a**,**b**) bar, 50 µm; and (**c**,**d**) bar, 10 µm.

The assay for CLP activities after all manipulations according to the Image-iT™ LIVE Green Poly Caspases Detection Kit protocol demonstrated that the Latr B treatment of the pollen tubes growing in vitro activated CLPs. Green fluorescence signals were observable in the pollen tubes of both the self-compatible petunia clone (Figure 9a,b) and the *S. lycopersicum* tomato cultivar Blush (Figure 9c,d).

An analysis of these results suggests that the disassembly of the actin cytoskeleton by Latr B both in vitro and in vivo activates CLPs.

### 3.6. Determining the Expression Level of IPT5, LOG, CKX1, and CKX2 Genes Involved in the CK Biosynthesis of Cytokinins

**Figure 10:**
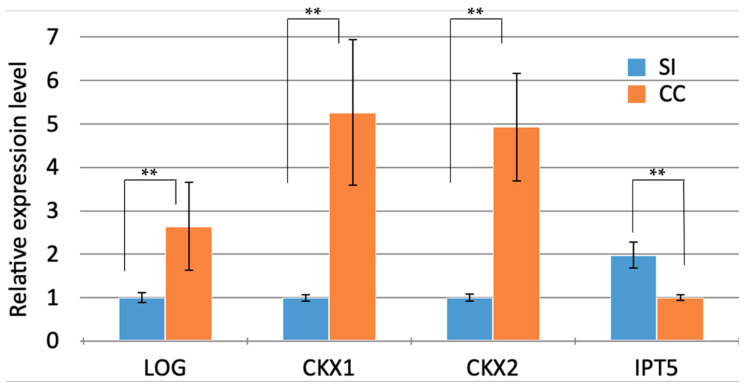
Expression of *LOG*, *CKX1*, *CKX2* and *IPT5* in the pistils (stigma and style) after cross-compatible (CC) and self-incompatible (SI) pollinations (6 h) according to RT-qPCR analysis (mean values ± standard deviations, Student’s *t*-test, ** *p* < 0.05).

According to the RT-qPCR analysis, the *IPT5* gene expression in self-incompatible pollination is two times higher than its expression in cross-compatible pollination (Figure 10).

The *LOG* expression level in self-incompatible pollination was slightly lower than its expression in cross-compatible pollination. However, the expression levels of *CKX1* and *CKX2*, which catalyze CK degradation due to the removal of the isoprenoid side chain, were much higher in cross-compatible pollination than in self-incompatible pollination. Thus, the high concentrations of CK in self-incompatible pollination are probably induced by the increased expression of the *IPT5* gene and the decreased expression of *CKX1* and *CKX2*, whereas the low concentrations of CK in the case of cross-compatible pollination are probably induced by the increased expression of *CKX1* and *CKX2*. 

## 4. Discussion

### 4.1. Self-Incompatibility Induced PCD

Pollination, which comprises a number of intercellular interactions, is one of the most significant stages in the developmental program of flowering plants [36]. Pollen tube growth is a complex event comprising numerous integrated processes (adhesion, hydration, pollen germination, and pollen tube growth), including the control mechanism limiting self-pollination, namely, self-incompatibility (SI) [37].

Characteristic of Solanaceae, Rosaceae, Papaveraceae, Poaceae, Commelinaceae, Ranunculaceae, and Plantaginaceae is a gametophytic SI, with the incompatibility phenotype determined by the genotype of the haploid pollen. Pollen is rejected when the S-haplotype of the haploid pollen corresponds to either S-haplotype of the diploid pistil [38,39,40,41]. SI may manifest itself in the stigma, style, or ovary, but it is believed that the inhibition in style tissues is more characteristic of the gametophyte SI [42]. In the majority of systems of gametophytic SI, the incompatible pollen successfully germinates on the stigma and grows into style tissues; however, the growth is then arrested, as is observed in Rutaceae [43] and Solanaceae [44].

Depending on the particular family, the male component of the F-box protein, referred to as SFB or SLF, is expressed in pollen. This was observed for the first time in Antirrhinum [45] and then in Prunus [46], Petunia [47], and Citrus [48]. Thus, the SI mechanism of Solanaceae utilizes S-RNase-based RNA degradation of the incompatible pollen tube; in this process, S-RNase acts as a female determinant and SLF (or SFB) acts as a male determinant. However, manifold other external and internal factors influence pollen rejection/acceptance [48].

Our studies have demonstrated the presence of PCD markers in the incompatible petunia pollen tubes such as DNA fragmentation, CLP activation, and the disintegration of the plasma membrane [22,23]. Transmission electron microscopy demonstrates a complete destruction of the pollen tube contents by 12 h after self-incompatible pollination [22].

Our results are insufficient to determine the type of SI-induced PCD in *P. hybrida*. The current classification of plant PCD comprises the main types of apoptosis like PCD, aging, and vacuole-mediated cell death [16]. We can only assume that our case is very similar to apoptosis-like PCD based on the disruption of membrane integrity, cytoplasm condensation, DNA fragmentation, and CLP activation [22].

The attempts to identify the genes involved in signal transduction during the response to SI with *Leymus chinensis* (Poaceae) as the object [48] via transcriptomic analysis revealed the signals associated with calcium (Ca^2+^), protein phosphorylation, phytohormones, reactive oxygen species, nitric oxide, cytoskeleton, and PCD.

The most recent genome-wide transcriptome studies show the global pattern of the PCD process involving thousands of PCD-associated genes. In particular, Hanamata et al. [49] examined the gene expression profile during PCD development in the tapetum cells of rice pistils, which included the specific transcription factors necessary for the degradation of tapetum and eighteen genes involved in the hormonal metabolism (four genes associated with IAA, two genes with ABA, nine genes with ethylene, one gene with CK, one gene with jasmonic acid, and two with gibberellin). This demonstrates that the genes coding for phytohormones are involved in this process. An analysis of petunia and arabidopsis transcriptomes suggested that hormones transcriptionally regulate the autophagy-related genes [50,51,52]; thus, phytohormones are most likely able to modulate autophagy via signal transduction [53].

The mechanism underlying the hormonal regulation of the polarized growth of male gametophytes is among the basic problems in plant sexual reproduction [48,54]. We have pioneered a demonstration of the involvement of hormonal regulation in the SI mechanism of *P. hybrida* [24]. However, the specific roles of individual phytohormones in the control of male gametophyte germination and growth are still vague. Nonetheless, our own data and published data suggest some hypotheses for the mechanism underlying this control.

One of the first responses to pollination is the synthesis of ethylene [24]. Pollination activates the expression of the genes coding for 1-aminocyclopropane-1-carboxylate (ACC) synthase and 1-aminocyclopropane-1-carboxylate oxidase (ACO), controlling the synthesis of ethylene [55]. The main site of ethylene synthesis in petunia is the stigma, and pollen germination is also accompanied by ethylene synthesis [55].

The pretreatment of self-incompatible petunia stigmas with aminoxyacetic acid (AOA, an inhibitor of ACC synthesis) before self-pollination stimulated the growth of pollen tubes, which displayed no signs of PCD. This favors the assumption that ethylene is among the first players in the control of the PCD progress in the incompatible pollen tubes in the course of S-RNase-based SI [22].

Different contents of IAA, ABA, and CKs in the stigma and style tissues are characteristics of the germination and growth of petunia pollen tubes after compatible and self-incompatible pollinations [24]. In particular, a high level of ACC in the stigma tissues is observable in the pollen tube growing along the stigma conductive tissues after a compatible pollination, as is as pronounced ACC synthase activity and an increase in IAA content in the stigma and style tissues. After a self-incompatible pollination, the pollen tubes’ growth is arrested in the style tissues and is accompanied by an intensive ethanol synthesis in the stigma tissues as well as drastic increases in the ABA content (in the stigma and style) and CKs in the style [24].

Thus, a phytohormone-dependent signal transduction is one of the important mechanisms underlying PCD in plants [53]. In addition, PCD can be triggered by a wide range of different signals, many of which induce cell differentiation and proliferation in other situations [51].

### 4.2. CK Inhibits Pollen Germination and Pollen Tube Growth in Solanaceae

Most frequently, CKs are regarded as hormones with a positive effect. They are able to stimulate the proliferation of plant cells and the transport of nutrients to cell and can slow down senescence, etc. [56].

The phytohormone-dependent signal transduction pathway is one of the important mechanisms underlying PCD in plants [57,58]. As has been recently shown, high CK levels induce PCD in both animal and plant cells [58,59,60], thereby unexpectedly revealing the role of this plant hormone. The addition of high doses of 6-BAP to a proliferating cell suspension of several plant species (including *Arabidopsis thaliana*, *Daucus carota*, and *Medicago truncatula*) slowed down cell growth and induced their death [61,62]. An analysis of distinctive features (DNA fragmentation, the condensation of nuclear chromatin, and the release of cytochrome c from mitochondria) revealed a programmed character of induced cell death [60]. Kaźmierczak et al. [63] describe the mechanism of kinetin-induced death of *Vicia faba* ssp. minor root cortex cells.

As for our experiments, the exogenous zeatin treatment inhibited petunia pollen tube growth both in vitro [27] and in vivo [23]. In this work, we have demonstrated that the exogenous zeatin treatment inhibits the germination and growth of the tomato pollen tubes both in vitro (Figure 6) and in vivo (Figure 3b; Table 2 and Table 3).

In a self-incompatible pollination, the pollen–pistil system of petunia displays a high level of endogenous CKs that reach a maximum 6–8 h after pollination at the moment of the self-incompatible pollen tubes’ growth arrest, particularly in the style, where this arrest takes place [24].

It is difficult to consider the role of an individual phytohormone in a process, and it is more adequate to speak about a hormonal balance in general, or at least about an interaction between a pair of phytohormones. In particular, it is known that CKs increase ethylene synthesis by influencing ACC synthase [64,65]. Many recent experiments suggest the involvement of ethylene in the induction of PCD [66,67]. In addition, phytohormones are able to affect the degradation intensity of CKs; for example, auxin accelerates degradation, whereas ABA inhibits this process [68,69].

### 4.3. CK Acidifies Pollen Tube Cytoplasm

According to the relevant published data [70], the transduction of hormonal signals in plant cells can include a transient shift in the cytoplasmic pH (PHc) towards either range. It is known that the intracellular pH value is an essential factor in the control of the key intracellular processes, such as gene expression, protein synthesis, and the remodeling of the cytoskeleton [71].

It has been reported that an increase in the intracellular pH is a necessary condition for the activation of a pollen grain during its germination [72]. Exogenous phytohormones are capable of transiently modulating the PHc in pollen grains [26]. The character of a hormone-induced PHc shift significantly depends on the physiological state of the male gametophyte. In the case of hydrated pollen grains, the PHc decreased but increased to a noticeable degree during their germination [73].

The PHc of petunia pollen grains germinating in vitro changed in response to the addition of phytohormones to the culture medium [30]. In particular, the addition of IAA and ABA to the medium alkalinized the cytoplasm by 0.4–0.5, whereas the addition of kinetin shifted the PHc into the acid range, down to 5.3–5.8 [30]. The PHc of a growing pollen tube is normally close to neutral (6.6–6.8).

SI drastically acidifies the pollen tube cytosol in Papaver [57] to a PHc of 5.5. The acidification of the pollen tube cytosol plays a key role in PCD in self-incompatible pollen tubes by creating the optimal conditions for the activation of caspase-3-like/DEVDase [29]. CK is the only phytohormone able to rather rapidly decrease the PHc during the first minutes after the treatment of petunia pollen tubes [30].

The fact that the content of CKs is several folds higher by the time the growth of self-incompatible pollen tubes is arrested when compared with a compatible pollination [24] and that the expression of the *IPT5* gene is high by the time the growth of self-incompatible pollen tubes is arrested (Figure 10) suggest that this phytohormone is also necessary in this high amount for a decrease in the PHc; perhaps this creates the optimal conditions for the function of CLPs.

The knowledge accumulated so far reveals the presence of an intricate, self-organizing SI-signaling network involving the tip-localized Rho GTPase, apical gradients of cytosolic Ca^2+^, reactive oxygen species, actin cytoskeleton, and vesicular trafficking delivering cell-wall and membrane materials to the growing pollen tubes [74]. The construction of transgenic arabidopsis lines with functional PrpS expressed in the pollen [75] and later, the functional transfer of both Papaver SI determinants to arabidopsis reproduction in planta [76,77], demonstrate that two components, PrpS and PrsS, are sufficient to induce an SI response in another species. This successful functional transfer implies that the components involved in the Papaver SI response are most likely rather ancient because they are able to function in highly divergent species [77]. Using genetically encoded fluorescent probes in combination with the imaging of living cells, Wang et al. [71] succeeded in confirming and assessing a number of early events of a decisive importance for the Papaver SI (an increase in the cytosolic Ca^2+^, a decrease in PHc, and the remodeling of actin cytoskeleton) as well as several later events (changes in the morphology of vacuoles and PCD).

As has been shown, the set of actin-binding proteins (ABPs) is important for the spatial distribution and dynamics of the cell actin cytoskeleton [78]. At least two among these ABPs are associated with the formation of the SI-induced actin foci, namely, actin-depolymerizing factor (ADF/cofilin) and cyclase-associated protein (CAP/Srv2p) [79]. Interestingly, the activities of these two ABPs are sensitive to PHc [80,81]. Thus, the SI-induced decrease in PHc can change the activities of these proteins.

### 4.4. CK Suppressed Actin Polymerization in Pollen Tubes

SI-induced acidification also plays a key role in actin remodeling in Papaver. The artificial manipulations of the PHc in pollen tubes demonstrate that a pH below 5.5 causes the formation of numerous F-actin foci. Buffering to PHc 7with the aim of preventing the SI-induced acidification blocked the formation of actin foci [57]. Changes in the cytoskeleton dynamics can influence the induction of PCD [82,83].

The importance of the cytoskeleton to the pollen tube growth cannot be overestimated. Pollen germination and the maintenance of polarized pollen tube growth require both temporal and spatial coordination of many cell functions, including the dynamic arrangement of the elements in the cytoskeleton, intracellular trafficking of the vesicles delivering cell-wall materials depending on exo- and endocytosis, the transmembrane transport of the main physiologically important ions, and transient changes in the parameters of intracellular ion homeostasis, such as PHc and Ca^2+^ [84,85]. The actin cytoskeleton provides the delivery of secreted substances and membrane vesicles to the apical zone of pollen tube owing to the activity of actin filaments during their polarized growth [78]. The remodeling of cytoskeleton structures at different stages of pollen tube growth implies that they respond to intracellular and extracellular signals [86].

Latr B, an inhibitor of actin, not only suppressed actin polymerization but also considerably influenced the distribution of microtubules in the *Nicotiana tabacum* pollen tube [87]. The actin cytoskeleton acts as the key system, providing the movement of organelles in the pollen tube [88]. In the pollen tubes of angiosperms, the organelles move forward to the tip along the actin cortical bundles and in the opposite direction along the central bundles according to the so-called reverse fountain pattern [89]. The destruction of microfilaments by Latr B arrested pollen tube growth. In this case, the movement of the vesicles was completely disorganized [90], interfering with cell wall construction.

The central role of the actin cytoskeleton of the male gametophyte in the regulation of germination is confirmed by the fact that Latr B, which inhibits actin, blocked both processes in petunia [29]. In addition, the content of endogenous IAA (the phytohormone playing a key role in the maintenance of polarized growth) drastically decreased during pollen tube germination and growth in the medium with a low Latr B content versus the content of CKs, which increased.

The changes in actin polymerization most likely represent a universal mechanism used by plants in PCD, although some details may differ.

The cultivation of petunia pollen in the kinetin-containing medium decreased the density of actin filaments along the entire pollen tube, a change which was most pronounced in its apical zone [29]. Kinetin (a CK) suppressed actin polymerization in pollen tubes, manifesting as a decrease in actin filaments down to 40%. The most pronounced decrease in the polymerized actin at a kinetin concentration of 10^−6^ was observed in the basal part. This effect of kinetin was in full accordance with its inhibitory effect on the pollen tube growth in petunia [23,24] and tomato (Figure 2c, Figure 3b and Figure 6; Table 1 and Table 2).

The action of S-RNase turned the actin cytoskeleton into point actin foci [91]; a similar picture is observed after the CK treatment of pollen tubes growing in vitro [30].

Latr B treatment influences CLP activities. Latr B pretreatment in vivo 2 h before pollination activates CLPs, which we have demonstrated in petunia via biochemical methods [39] and through intravital CLP imaging in petunia and tomatoes (Figure 8). Presumably, the inhibitory effect of CK treatment on the pollen tube germination and growth results from the destruction of the actin cytoskeleton.

However, taking into account that the Latr B treatment of pollen tubes growing in vitro (that is, when the actin cytoskeleton is destructed) increases the endogenous content of CKs. The chain of events most likely appears as follows: CKs → decrease in PHc → disassembly of actin cytoskeleton → CLP activation.

### 4.5. CK Stimulates CLP Activities

The degenerative stage of apoptosis begins from caspase activation [3]. As has been shown, the disassembly of apoptotic cells is an ordered and strictly controlled process organized by CLPs and the remodeling of cytoskeletal framework [91].

In *P. hybrida*, we observed different degrees of caspase-3-like/DEVDase activation in the pollen–pistil system depending on the type of pollination [22]. In particular, a flat, low level of CLP activity was observed after a self-compatible pollination during 24 h of pollen tube growth. On the contrary, caspase-3-like/DEVDase activity after a self-incompatible pollination was quite different, drastically increasing 2 h after pollination, followed by a further approximately fivefold increase (compared with self-compatible pollination). The DEVDase activity remained high for 2 h (4 h after pollination) and decreased during the next 2–4 h, that is, when the self-incompatible pollen tube growth was arrested because of PCD [22]. These data suggest that the activation of caspase-3-like protease is a factor of S-RNase-based SI-induced PCD in *P. hybrida*.

The zeatin (10 µM) pretreatment of the petunia and tomato stigmas 2 h before compatible pollination stimulated CLP activities in pollen tubes (Figure 4 and Figure 5) and considerably inhibited pollen tube growth in vivo (Table 2 and Table 3). A similar effect of CLP activation is observable in self-incompatible pollination.

Thus, the phytohormone CK plays a decisive role in the arrest of pollen tube growth in the case of RNase-type SI by activating CLPs, which are the determinants of PCD in the petunia and tomato male gametophytes.

### 4.6. CK Biosynthesis Genes Activity

Several gene families play key roles in the maintenance of CK homeostasis: isopentenyltransferase (*IPT*) catalyzes the limiting stage in the de novo formation of CKs, and CK riboside 5′-monophosphate phosphoribohydrolase gene (*LOG*) encodes an enzyme that catalyzes the phosphoribohydroxylation of CK nucleotides for the synthesis of biologically active nitrogenous bases of CK and CK oxidase/dehydrogenase (*CKX*), which irreversibly activate CKs by cleaving the N6 side chain [33,56,92].

Currently, the enzymes controlling all stages of CK biosynthesis in plants have been isolated, and the corresponding genes have been identified. The first stage in CK biosynthesis is the synthesis of isopentenyl nucleotides from ATP or ADP and dimethylallyl pyrophosphate (DMAPP); it is catalyzed by the isopentenyltransferase enzyme [93,94].

According to recent data, different stages of CK biosynthesis are implemented in different plant tissues. The main site of the synthesis of CK nucleotides is the root tip; a certain amount is also synthesized in the shoot apex, flowers, and fruits [56]. Along the xylem, CK nucleotides are delivered to the shoot apex, which is the main site at which free CKs are synthesized [56,95]. Different subcellular localizations of IPT suggest that the biosynthesis can take place in the cytosol, chloroplasts, and mitochondria [96]. This stage of biosynthesis is the limiting one at which the CK concentration in plant tissues is controlled. A change in the *IPT* gene expression level has a most pronounced effect on the content of CKs [56,97].

Our initial data on the endogenous content of phytohormones during petunia compatible and self-incompatible pollinations [24] suggested to us that expression of the genes coding for CKs could increase at the moment at which the self-incompatible pollen tube growth was arrested. Our assumptions emerged to be true: the expression of the *IPT5* gene is doubled when compared with its expression in a compatible pollination (Figure 10). However, the *LOG* expression level in self-incompatible pollination was slightly lower than its expression in cross-compatible pollination. At the same time, the expression levels of *CKX1* and *CKX2*, which catalyze CK degradation due to the removal of the isoprenoid side chain, were much higher in cross-compatible pollination than in self-incompatible pollination. Thus, high concentrations of CK in self-incompatible pollination are probably induced by an increased expression level of the *IPT5* gene and decreased expression levels of *CKX1* and *CKX2*, whereas low concentrations of CK in the case of cross-compatible pollination are probably induced by increased expression levels of *CKX1* and *CKX2*. 

Thus, the following sequence of events is outlined: pollination (SI induction) leads to an increase in CKs, which shifts the PHc to the acid range (to 5.5–5.8), followed by the disorganization of cytoskeleton to point foci with the subsequent activation of CLPs, triggering the remaining PCD responses.

### 4.7. CK as a Factor of SI-Induced PCD

A characteristic of the solanaceous-type SI mechanism is S-RNase-based RNA degradation in the incompatible pollen tube; note that S-RNase serves as a female determinant, while SLF or SFB serve as male determinants. The self-recognition of female and male determinants activates the network of intracellular signals in the incompatible pollen tube, leading to the slowdown of its growth and eventually to PCD.

CKs play a decisive role in this signaling and have a polyfunctional effect. Herein, the following pattern of a solanaceous-type SI is outlined. CKs act at several stages (boldfaced).

(1) S-RNases are extracellular proteins that accumulate in the cells of the style conductive tract and are secreted into the extracellular space of the tract [98]. These proteins are expressed exclusively in the pistil, mainly in the upper style part where the growth of incompatible pollen tubes is arrested [97]. S-RNases act as highly specific cytotoxins [16].

(2) SFB or SLF (depending on the family) are expressed in pollen [43,46]. The S-haplotype in Solanaceae comprises a set of 16–20 SLF genes, which jointly enhance the SI function in pollen [13,99]. As has previously been assumed, these derivatives of the pollen S-gene serve either as S-RNase inhibitors or S-RNase receptors [100].

(3) S-RNase is not the only enzyme responsible for pollen rejection. Certain modifier genes are also indispensable for the SI response [14,101].

(4) In the case of a compatibility response, SLF proteins under a certain S-haplotype enhance the ubiquitination and degradation of all foreign S-RNases except for their own. Their own S-RNases enhance the rejection of the pollen tube to avoid degradation [99].

(5) The theory of substitute S-RNase compartmentalization implies that S-RNases are isolated in membrane compartments which prevent their cytotoxic effect in the cytosol of compatible pollen tubes. On the contrary, the vacuole is destroyed in the incompatible pollen tubes, releasing S-RNases into the cytosol [102]. Before the vacuole is destroyed, several important physiological steps take place.

An alternative model—the general inhibitor model—is proposed. It is assumed that the Prunus pollen S-determinant inhibits all RNases except for the genetically identical one [15]. The Prunus SLF proteins act as blockers, protecting their own S-RNase from degradation by SLF- and SFB-like proteins, thereby enhancing the S-RNase’s activity and the arrest of pollen tube growth [103].

(6) The self-recognition of female and male determinants activates the network of intracellular signals in the incompatible pollen tube, eventually leading to PCD. The cytosolic Ca^2+^ concentration increases, followed by its decrease [104,105].

(7) The contents of reactive oxygen species and nitric oxide increase [106,107].

(8) **CKs** are synthesized in large amounts.

(9) The PHc decreases to 5.6–5.8 (**CKs**).

(10) The actin cytoskeleton depolymerizes and remodels to point foci (**CKs**) [105,106].

(11) The actin foci co-localize with special actin-binding proteins [71].

(12) The activation of CLPs (**CKs**) occurs.

According to some data, MAP kinase activates DEVDase and remodels actin cytoskeleton [108].

(13) The degradation of actin cytoskeleton interferes with the integrity of the plasma membrane and possibly destructs the vacuole, thereby releasing S-RNases.

(14) The degradation of nuclear DNA, the degradation of cell organelles, and PCD occur.

Our analysis demonstrates that CLPs and the actin cytoskeleton are involved in SI-induced PCD in petunia. Thus, the basic question as to whether the cytoskeleton is the target or an active player in SI-induced PCD remains to be clarified. Evidently, these results reflect the nonuniformity of the signaling pathways.

The research into the SI phenomenon has been relevant for more than several decades. New plant species with SI are discovered, for example, pomelo, a Citrus species [43]. It has been shown that artificially induced SI is able to work not only in reproductive organs but also in vegetative cells [108]. Self-compatibility and SI have their own advantages and disadvantages for each individual crop and the aims of its cultivation. The ability to change SI to self-compatibility and vice versa is currently an important demand; different mechanisms, both physiological [109,110,111,112,113] and molecular–genetic [11,113,114,115], are already used for this purpose.

## 5. Conclusions

The results of this work show that endogenous CKs are necessary in arresting the growth of incompatible pollen tubes during the functioning of the S-RNase-type SI mechanism and trigger SI-induced PCD. the exogenous treatment of petunia and tomato stigmas with zeatin at all concentrations tested resulted in complete blockage and a significant inhibition of male gametophyte growth in the cases of self-incompatible and compatible pollinations, respectively. The inhibitory effect of CKs were also manifested when zeatin was added to the culture medium for growing pollen and pollen tubes. *In vitro* pollen germination on kinetin-containing culture medium led to a decrease in the density of actin filaments along the entire pollen tube, as previously described. Additionally, CKs inhibit pollen tube growth due to acidification of the cytoplasm and the disorganization of the actin cytoskeleton (AC).

According to our hypothesis, CKs at high concentrations acidify the cytosol SI pollen tubes, thus creating favorable conditions for CLP activation and perhaps for AC reorganization. High concentrations of exogenous CKs resulted in the activation of CLPs in vivo. Correspondingly, CKs in combination with CLPs and S-RNase trigged the AC destruction of SI pollen tubes, the disruption of the membrane integrity and organelles, and the eventual degradation of DNA as the final stage of the S-RNase-based SI-induced PCD in Solanaceae.

## Figures and Tables

**Table 1:** List of the primers used for qRT-PCR.

Primer	Sequence (5’➝3’)
IPT5f	GAATCCGACGGTCCATTGGT
IPT5r	GCTGGAAAATGGGTGGCAAG
Ef1af	CCTGGTCAAATTGGAAACGG
Ef1ar	CAGATCGCCTGTCAATCTTGG

**Table 2:** Effects of zeatin pretreatment (before self-pollination) on the length of *S. lycopersicum* cultivar Blush pollen tubes (PTs).

Time after Pollination	Self-Compatible Pollination (Control), Length of PTs (μm)	Self-Compatible Pollination + Zeatin (10 µM), Length of PTs (μm)
2 h	1345 ± 68	250 ± 50
4 h	2840 ± 105	480 ± 35
6 h	5488 ± 324	1278 ± 87
24 h	Reached the ovary	2898 ± 176

**Table 3:** Effects of zeatin pretreatment of stigmas on the pollen tube growth in *S. chilense.* PTs, pollen tubes.

Time after Pollination	Self-Incompatible Pollination (Control), Length of PTs (μm)	Self-Incompatible Pollination + Zeatin, Length of PTs (μm)
2 h	1298 ± 45	0
4 h	1601 ± 59	0
6 h	1691 ± 87	0
24 h	1898 ± 102	0

## Data Availability

Not applicable.

## Supplementary Materials

The following supporting information can be downloaded at: https://www.mdpi.com/article/10.3390/biom13071033/s1, Video S1: Fluorescence microscopy of the petunia pollen tube growing in vivo, using an Image-iT™ LIVE Green Poly Caspases Detection Kit. Cross-compatible pollination (+zeatin pretreatment). The green fluorescence signal in the pollen grains and tubes indicates the presence of CLP activity in cells.
